# Primary Peritoneal Angiosarcoma Metastatic to Liver and Bone without History of Radiation Therapy

**DOI:** 10.1155/2018/1257284

**Published:** 2018-01-09

**Authors:** Way Chiang, Zofia Tynski

**Affiliations:** ^1^Department of Family Medicine, Clarion Hospital, 1 Hospital Drive, Clarion, PA 16214, USA; ^2^Department of Pathology, Clarion Hospital, 1 Hospital Drive, Clarion, PA 16214, USA

## Abstract

Angiosarcoma is a rare vascular soft tissue tumor of endothelial origin most commonly seen in the elderly as a primary cutaneous head and neck malignancy. Furthermore, a peritoneal angiosarcoma is an exceedingly rare entity. This is the second case of primary peritoneal angiosarcoma reported in literature that is not associated with prior radiotherapy. Herein, we describe a case of primary peritoneal angiosarcoma metastatic to both the liver and bone in a male patient with metachronous renal cell carcinoma and parathyroid adenoma.

## 1. Introduction

Vascular sarcomas are a subset of soft tissue sarcomas which include angiosarcomas, hemangiosarcomas, and lymphangiosarcomas. These comprise approximately 4% of all soft tissues sarcomas. The most commonly stated primary sites of involvement include the viscera, retroperitoneum, head and neck, and extremities [1]. Several exposures have been proposed as risk factors, including vinyl monomers, liver exposure to Thorotrast, arsenic, androgenic anabolic steroids, trauma, chronic lymphedema, and radiotherapy, but the majority of reported cases have no known exposures [1, 2]. A literature search for case reports of primary peritoneal angiosarcoma in English was performed revealing five published cases associated with radiotherapy, one published case without any known exposure, and none with a history of two metachronous primary neoplasms and no history of radiotherapy [3–8].

Primary peritoneal angiosarcoma is a malignancy of endothelial origin and a rare primary site compared to the more typical cutaneous origins on the head or extremities, which are better described in the literature. Their characteristics are similar however, with best agreed upon treatment being resection of localized malignancy ideally, though with high failure rate through emergence of distant metastasis within months despite generous surgical margins [1]. Prognosis is typically poor and the malignancy is invariably fatal. The case described is an unusual case involving emergence of primary peritoneal angiosarcoma approximately two years after successful robotic surgical resection of clear cell type renal carcinoma and approximately one year after resection of a parathyroid adenoma.

## 2. Case Presentation

The patient was a 59-year-old male former smoker with a past medical history of stage 1-2/4 type I left sided renal cell carcinoma status after robotic partial nephrectomy, parathyroid adenoma status after resection, history of probable idiopathic thrombocytic purpura status after splenectomy, hypertension, diabetes mellitus type 2, coronary artery disease with past myocardial infarction and stent placement, and internal pacemaker-defibrillator placement. The patient had begun complaining of progressive fatigue, arthralgias, myalgias, and back pain beginning approximately one year before diagnosis at his primary care provider's office, which initially seemed to be associated with his, at the time, recent parathyroid resection. Lyme's disease, endemic in the patient's geographic region and often considered in nonspecific presentations with these symptoms, was ruled out by antibody assay. On retrospective analysis of the patient's history, he apparently had worked at a local university in maintenance and did not have a known history of employment in manufacturing or significant chemical handling. There was no known family history of genetic conditions that would predispose the patient to multiple malignancies.

**Figure 1:**
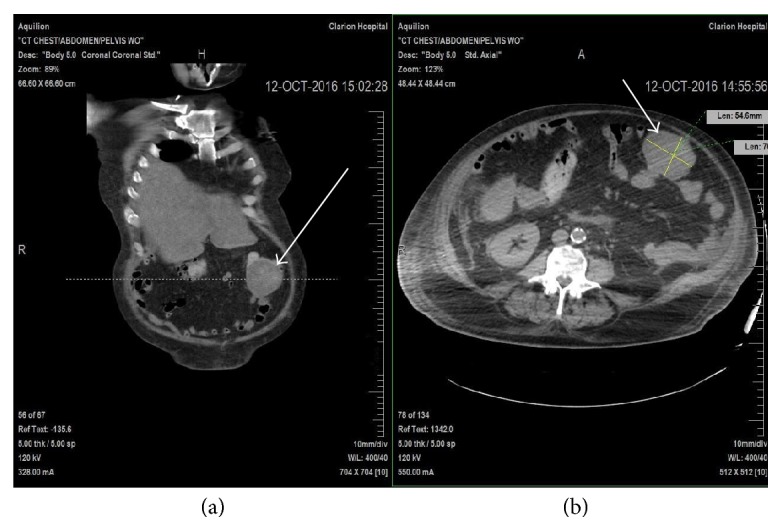
(a) Coronal view of noncontrast CT demonstrating left abdominal wall mass (arrow). (b) Axial view of noncontrast CT demonstrating left abdominal wall mass measuring approximately 7 cm × 5.5 cm (arrow).

The patient progressively worsened over months; he developed nausea, dry heaving, and decreased appetite. CT of the chest and HIDA scan had been performed which revealed nonobstructive cholelithiasis with normal gallbladder function. The patient subsequently underwent a noncontrast CT of abdomen and pelvis, which revealed a large abdominal mass measuring 7 cm × 5.5 cm abutting the mid portion of the transverse colon, with numerous metastatic foci in the liver, lymphadenopathy adjacent to the pancreatic head, a large lytic lesion involving the tenth rib, and scattered lytic areas throughout the spine and left iliac wing (Figure 1). The patient then underwent core biopsy of the abdominal mass.

Initial core biopsy of the abdominal mass revealed highly vascular tissue, the majority necrotic, with small atypical cells seen on frozen section. Immunohistochemical staining for RCC was negative. CD10 was equivocal with chromogranin A and podoplanin (D240) both being negative. Vimentin was strongly positive. A consult was made, an additional staining including Cam 5.2, CD31, and ERG. Cam 5.2 revealed rare mesothelial cells and the vascular proliferation was positive for both CD31 and ERG. It was concluded that the degree of necrosis precluded a definitive diagnosis but that a vascular neoplasm or well-differentiated angiosarcoma could not be excluded.

**Figure 2:**
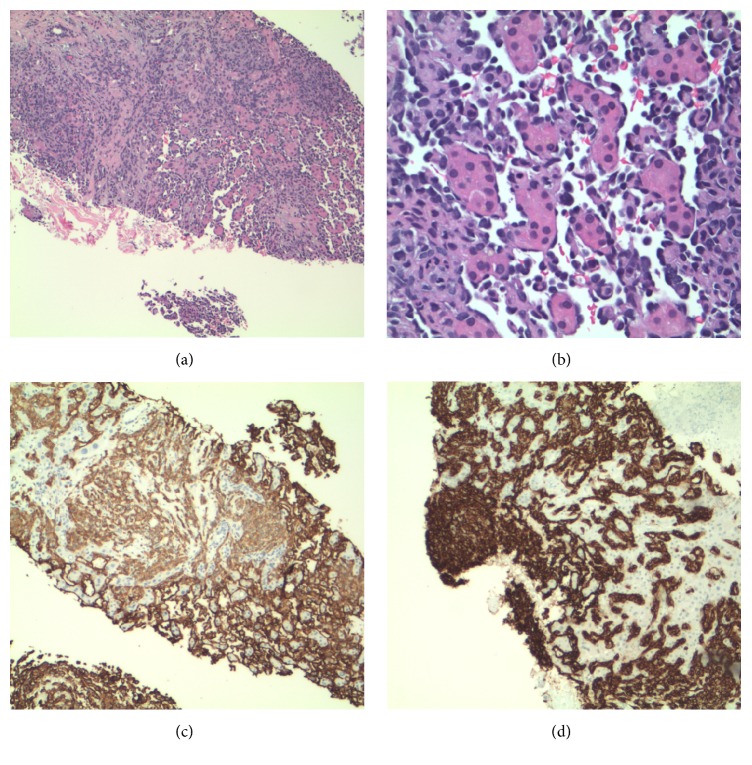
(a) 100x angiosarcoma H&E demonstrating tumor with high cellularity and prominent vascular network. (b) 400x angiosarcoma H&E demonstrating vasculature lined by plump, pleomorphic, endothelial cells. (c) 100x demonstrating CD31 positivity. (d) 100x demonstrating CD34 positivity.

**Figure 3:**
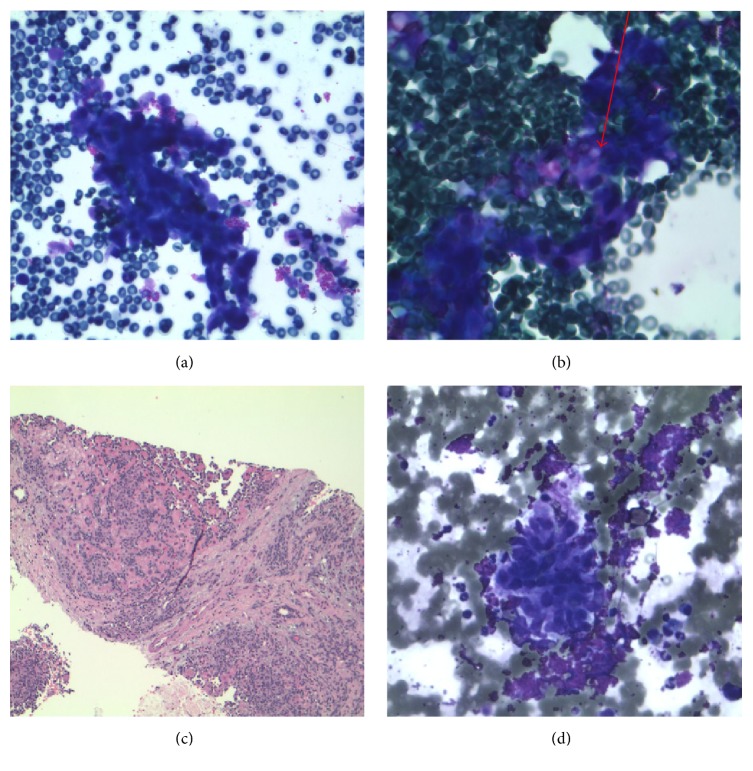
(a) 400x Romanowsky stain demonstrating epithelioid cluster delineating vasoformative features. (b) 630x Romanowsky stain demonstrating vessel and plump hyperchromatic nuclei and intracytoplasmic lumen (arrow). (c) 100x angiosarcoma H&E demonstrating tumor consisting of anastamosing vascular channels and hobnailing nuclei. (d) 400x Romanowsky stain demonstrating malignant spindle cellular cluster.

Repeat core liver biopsy and immunohistochemical staining were performed at an outside institution. The liver biopsy delineated a very hypercellular specimen (Figure 2(a)). The tumor was comprised of a prominent vasoformative network (Figure 2(b)). The endothelial lining cells were plump and pleomorphic. They had high nuclear to cytoplasmic ratio with hobnailing hyperchromatic nuclei. The cytoplasm was eosinophilic with indistinct cell borders. The cytology delineated a hypercellular aspirate (Figure 3). The cells formed a tightly cohesive cluster of epithelioid cells. The cells harbored hyperchromatic nuclei with eosinophilic cytoplasm. On occasion, the cytoplasm demonstrated cytoplasmic vacuoles and intracytoplasmic lumina. Focally, the clusters were composed of very atypical spindle cells. The cytology specimen stained negative for cytokeratin AE1/AE3 and positivity for CD31 and CD34. Since the specimen stained for two vascular immunohistochemical stains (CD31 and CD34) in conjunction with pancytokeratin negativity, a diagnosis of angiosarcoma was rendered (Figures 2(c) and 2(d)).

The patient was scheduled for systemic chemotherapy with doxorubicin as well as palliative, single modality radiation therapy of the spine. Radiation was completed first. The patient's chemotherapy regimen was changed to taxol rather than doxorubicin due to low ejection fraction seen on MUGA scan. However, prior to initiation of chemotherapy the patient was hospitalized for confusion, lethargy, and enterococcal bacteremia. The decision was made to discharge the patient with hospice care and he died 13 days later.

## 3. Discussion

Angiosarcoma is a rare subtype of sarcoma characterized by endothelial cell proliferation, most frequently occurring in the elderly [2]. It may arise anywhere in the body, but cutaneous head and neck lesions are seen most commonly. Immunohistochemical staining generally includes positivity for CD31, CD34, factor VIII, agglutinin 1, and VEGF [9]. An ERG, a newer vascular stain, usually demonstrates nuclear positivity. Presentation is nonspecific, though it has been suggested that malignant ascites is the most frequently seen manifestation [6]. The hepatic variant of angiosarcoma is best known for its relationship to chemical exposures but remains a rare entity that can be mistaken for atypical hepatocellular carcinoma [2]. To our knowledge, the patient described did not experience significant ascites but did develop anasarca later in his disease.

The differential diagnosis in this patient is renal cell carcinoma (RCC), unclassified for which he underwent a partial nephrectomy two years ago. The clear cell is the most common type of RCC (CCRCC), which usually originates in the proximal convoluted tubule. Assuming that the patient had the most common type of RCC, the morphology seen is very distinct. The architecture of RCC is either compact (sheets), alveolar, or tubulocystic. The tumor cells are large, polygonal with ample clear cytoplasm. The clear cell cytoplasm is indicative of lipid or glycogen accumulation. The cells possess delicate but distinct cell membranes. The cells are permeated by a thin wall vasculature reminiscent of “chicken wire” with nuclei that are rather small. Nucleoli are variably noted.

Angiosarcoma, as the name implies, has a prominent vascular component. However, the vascular network present in angiosarcomas is that of plump and pleomorphic endothelial cells, rather than a thin wall vasculature present in CCRCC. The nuclei of the endothelial cells impart a hobnailing configuration. In addition, the cytoplasm is eosinophilic in angiosarcomas as opposed to clear in CCRCC. However, both angiosarcomas and CCRCC can have an alveolar architecture. Furthermore, the immunohistochemistry can be utilized to separate these two malignancies. Generally speaking, vascular and sarcomatous stains are positive in angiosarcomas but not keratin stains. The vascular markers utilized to diagnosis this patient were CD31, CD34, and ERG. Vimentin, a mesenchymal marker, was also positive. However, this is a nonspecific marker which is also positive in CCRCC. Since CCRCC is an epithelial malignancy, keratins are positively expressed. The CAIX, PAX8, PAX2, CD10, and RCC demonstrate restricted expression in CCRCC but not in angiosarcomas.

Attempts have been made to compare radiation associated angiosarcomas with spontaneous angiosarcomas in case reviews. It is proposed that the two are ultimately very similar in histology, in disease pattern, and in mutation profile [4]. Other literature has found that MYC gene amplification may be more distinct for postradiation malignancy compared to primary malignancy [7]. This difference, however, does not appear to significantly impact the clinical disease course. Guo et al. found that postradiation angiosarcoma cells with high expression of MYC still showed low expression of the MAX mRNA product in the normal MYC/MAX heterodimerization pathway. Consistent with this was lack of effect on cell growth with pathway inhibition [10]. This suggests that other mechanisms are likely involved in tumorigenesis, though it is clear that radiotherapy or subsequent lymphedema represents significant risk factors for secondary disease.

Traditional treatment of soft tissue sarcomas consists of the acronym MAID: mesna, adriamycin (doxorubicin), ifosfamide, and dacarbazine. Radiotherapy is frequently used between cycles. Meta-analysis by Verma et al. examined outcomes of ifosfamide containing regimens versus non-ifosfamide-containing regimens, finding that though the addition of ifosfamide did appear to improve tumor response rate, there was not a significant improvement in 1-year survival [11]. Given the rarity of sarcoma in general, doxorubicin as monotherapy or in combination with other agents appears to remain preferable and best studied. Antiangiogenic therapies such as thalidomide have also been shown to induce objective response [12].

Data comparing treatment modalities for angiosarcoma specifically is complicated by small sample sizes. The chemotherapeutic agent of choice for soft tissue sarcomas remains doxorubicin, though paclitaxel has also been studied as well with response [13]. In the ANGIOTAX study, which consisted of patients with both metastatic and locally advanced disease, paclitaxel compared favorably to doxorubicin. Unfortunately the median survival remained only 7.6 months [12]. Review of literature specific to cutaneous primary sites suggests a median survival of 7 months with current treatment modalities [9]. Similar to aforementioned studies of MAID, it would appear that gains in tumor response with current treatment modalities have not improved survival rates to large degree.

The rarity of angiosarcomas, both after radiation and de novo, precludes strong evidence for a specific oncogenetic pathway or specific treatment strategy. Clinical detection and management is challenging given that cases will typically present in an advanced stage with aggressive progression. This is a particularly unusual case with de novo malignancy of the peritoneum in a middle aged patient with a history of two previous primary malignancies and no known exposures. This patient presented with metastatic disease not amenable to surgical resection and underwent radiotherapy before succumbing to his disease.
